# An in-silico modeling approach to separate exogenous and endogenous plasma insulin appearance, with application to inhaled insulin

**DOI:** 10.1038/s41598-024-61293-y

**Published:** 2024-05-13

**Authors:** Agnese Piersanti, Giovanni Pacini, Andrea Tura, David Z. D’Argenio, Micaela Morettini

**Affiliations:** 1 Department of Information Engineering, Università Politecnica Delle Marche, Via Brecce Bianche 12, Ancona, Italy; 2 Padua, Italy; 3 CNR Institute of Neuroscience, Padua, Italy; 4 Department of Biomedical Engineering, University of Southern California, Los Angeles, CA USA

**Keywords:** Biomedical engineering, Diabetes

## Abstract

The aim of this study was to develop a dynamic model-based approach to separately quantify the exogenous and endogenous contributions to total plasma insulin concentration and to apply it to assess the effects of inhaled-insulin administration on endogenous insulin secretion during a meal test. A three-step dynamic in-silico modeling approach was developed to estimate the two insulin contributions of total plasma insulin in a group of 21 healthy subjects who underwent two equivalent standardized meal tests on separate days, one of which preceded by inhalation of a Technosphere^®^ Insulin dose (22U or 20U). In the 30–120 min test interval, the calculated endogenous insulin component showed a divergence in the time course between the test with and without inhaled insulin. Moreover, the supra-basal area-under-the-curve of endogenous insulin in the test with inhaled insulin was significantly lower than that in the test without (2.1 ± 1.7 × 10^4^ pmol·min/L vs 4.2 ± 1.8 × 10^4^ pmol·min/L, p < 0.01). The percentage of exogenous insulin reaching the plasma, relative to the inhaled dose, was 42 ± 21%. The proposed in-silico approach separates exogenous and endogenous insulin contributions to total plasma insulin, provides individual bioavailability estimates, and can be used to assess the effect of inhaled insulin on endogenous insulin secretion during a meal.

## Introduction

Endogenous insulin secretion is sustained by pancreatic β cells to maintain blood glucose levels within a narrow range and its impairment is a prerequisite for the development of hyperglycemia characterizing diabetes mellitus in its different forms, mainly type 1 (T1D) and type 2 (T2D)^1^. Exogenous insulin administration may help to counteract this condition and, it represents the primary and lifesaving treatment for T1D^2^ and is used in T2D when oral antidiabetic medications fail to achieve adequate glucose control^3^. Despite its impairment, the endogenous component may contribute to the measured total plasma insulin in a non-negligible amount with respect to exogenous insulin. Therefore, the correct quantification of this variable insulin secretory capability may impact decisions regarding adequate exogenous insulin administration, fostering precision treatment^4–7^. Quantification of endogenous insulin secretion is also necessary in clinical trials for drug development. In fact, when testing pharmacokinetic properties and dose ranging for safety of new exogenous insulin formulations, early phase clinical trials are performed in healthy individuals, who have a preserved endogenous secretion.

Model-based approaches are established tools in the field of drug development to describe typical profiles of insulin concentration over time following exogenous administration of various insulin formulations^8,9^. However, issues related to the quantification of endogenous insulin secretion were often ignored since suppression of this component can be obtained through experimental procedures (e.g., hyper-insulinemic clamp or somatostatin administration), which, however, are complex to perform, present drawbacks for the individual and may be not fully effective in achieving adequate suppression^10^. When experimental conditions are more physiological and thus do not involve endogenous insulin suppression (as in mixed meal tolerance test), simple “baseline correction” methods are exploited that assume a single value of plasma glucose, insulin or C-peptide reflecting the endogenous component and correct the total plasma insulin for this constant quantity^11–13^. Plasma C-peptide is commonly considered a more reliable marker with respect to insulin since it is co-secreted with insulin in equimolar amount, but it is not extracted to a significant extent by the liver. Baseline correction, however, represents a very rough quantification as it does not reflect the dynamic changes during the test. Some attempts to provide a more refined correction based on C-peptide were made that involve mixed effects regression equations^14^ but such black box data-driven approaches disregard the description of the underlying physiological processes; moreover, robustness of such approaches in individual estimation are strongly affected by statistical assumptions including the choice of covariates^15^.

Dynamic model-based approaches relying on C-peptide are well-recognized methods to estimate endogenous insulin secretion. These approaches, however, these approaches were never exploited when an exogenous and endogenous insulin components overlap, as is the case in physiological conditions such as a mixed meal tolerance test. For this reason, a dynamic model-based approach that overcomes the limitations of the approaches previously proposed for separating exogenous from endogenous component is needed. Accordingly, the aim of the present study was to develop a dynamic in silico modeling approach that separates and quantifies the contributions of exogenous and endogenous insulin to measured plasma insulin under physiological conditions (i.e., meal tolerance test).

Although insulin is typically delivered by subcutaneous injection, alternate routes of administration are also used including pulmonary delivery via inhalation. In addition to less burden to the patient, inhaled insulin has demonstrated advantages compared with subcutaneous insulin in treating patients with T1D and T2D^16,17^. These include a faster onset of action, a more rapid return to baseline, a reduction in the incidence of hypoglycemia and improvement of glycemic control^18–20^. As a secondary aim, application of the proposed methodology to inhaled insulin administration was pursued to assess its effects on endogenous insulin secretion.

## Materials and methods

### Experimental protocol

**Figure 1:**
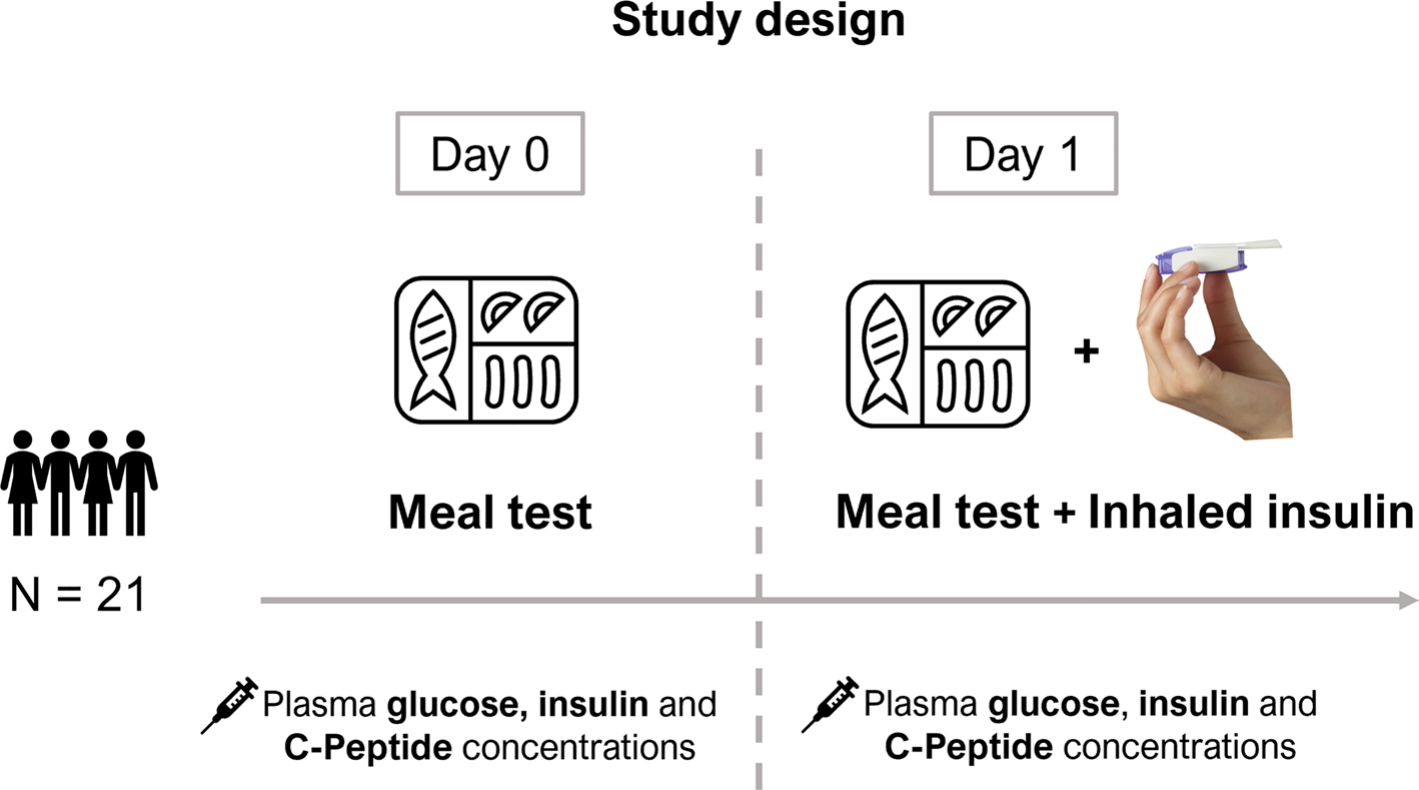
Graphical representation of the study design. Subjects underwent a standardized meal test on day 0 and a standardized meal test followed by Technosphere^®^ Insulin inhalation on day 1; plasma glucose, insulin and C-peptide blood samples were collected on both days.

A phase 1, single-center, open-label, randomized, crossover study in 21 healthy subjects (who provided written informed consent) was conducted upon approval of the institutional ethics committee (protocol number MKC-TI-141) and following the principles of the Declaration of Helsinki^14^. In summary, all subjects underwent two equivalent standardized meal tests (approximately 600 kcal to be completed within 20 min) on separate days after overnight fasting, with one of the tests preceded by inhalation of a dose (22U or 20U) of Technosphere^®^ Insulin through the Gen2B Inhaler. Inhalation maneuver training was performed using the BluHale™ Inspiratory Screening System (a proprietary experimental system developed by MannKind Corporation used to capture pressure–time profiles that are transmitted in real time to a graphical user interface that enables subjects to achieve prescribed inhalation effort parameters). Blood samples were analyzed for glucose, insulin, and C-peptide obtained over a 6-h period (0, 7, 15, 30, 60, 120, 240, 300, 360 min), thus providing one set of experimental data for the meal test (${G}_{MT}\left({t}_{i}\right),{I}_{MT}\left({t}_{i}\right),{CP}_{MT}\left({t}_{i}\right)$) and one set for the meal test with inhaled insulin (${G}_{MT+I}\left({t}_{i}\right),{I}_{MT+I}\left({t}_{i}\right),{CP}_{MT+I}\left({t}_{i}\right)$), with *i* indicating the i-th measurement time sample. Assays were performed by Bio Analytical Research Corporation (BARC, Lake Success, NY); electrochemiluminescence immunoassay (ECLIA, CV 4–5%) and competitive chemiluminescence immunoassay (CV 8–9%) were used for insulin and C-Peptide, respectively. The study design is summarized in Fig. 1 and the subjects’ demographic and basic characteristics are shown in Table 1.

**Table 1:** Subject’s demographic and basic characteristics.

Subject characteristics	Total (n = 21)
Gender^a^	10 M, 11 F
Age (years)	28.5 ± 8.6
Body weight (kg)	76.8 ± 13.5
BMI (kg/m^2^)	26.5 ± 3.0
Ethnicity	14 C, 3 A, 2 AA,1 H, 1 AI
G_b_ (mg/dL)	89.0 ± 6.7
I_b_ (µU/mL)	7.9 ± 4.8
Cp_b_ (ng/mL)	1.3 ± 0.6

Data are mean ± standard deviation unless otherwise indicated.

M: Male, F: Female, C: Caucasian, A: Asian, AA: African American, H: Hispanic, AI: American Indian, G_b_: Fasting glucose, I_b_: Fasting insulin, Cp_b_: Fasting C-peptide.

^a^Ascertained by self-report.

### Modeling

**Figure 2:**
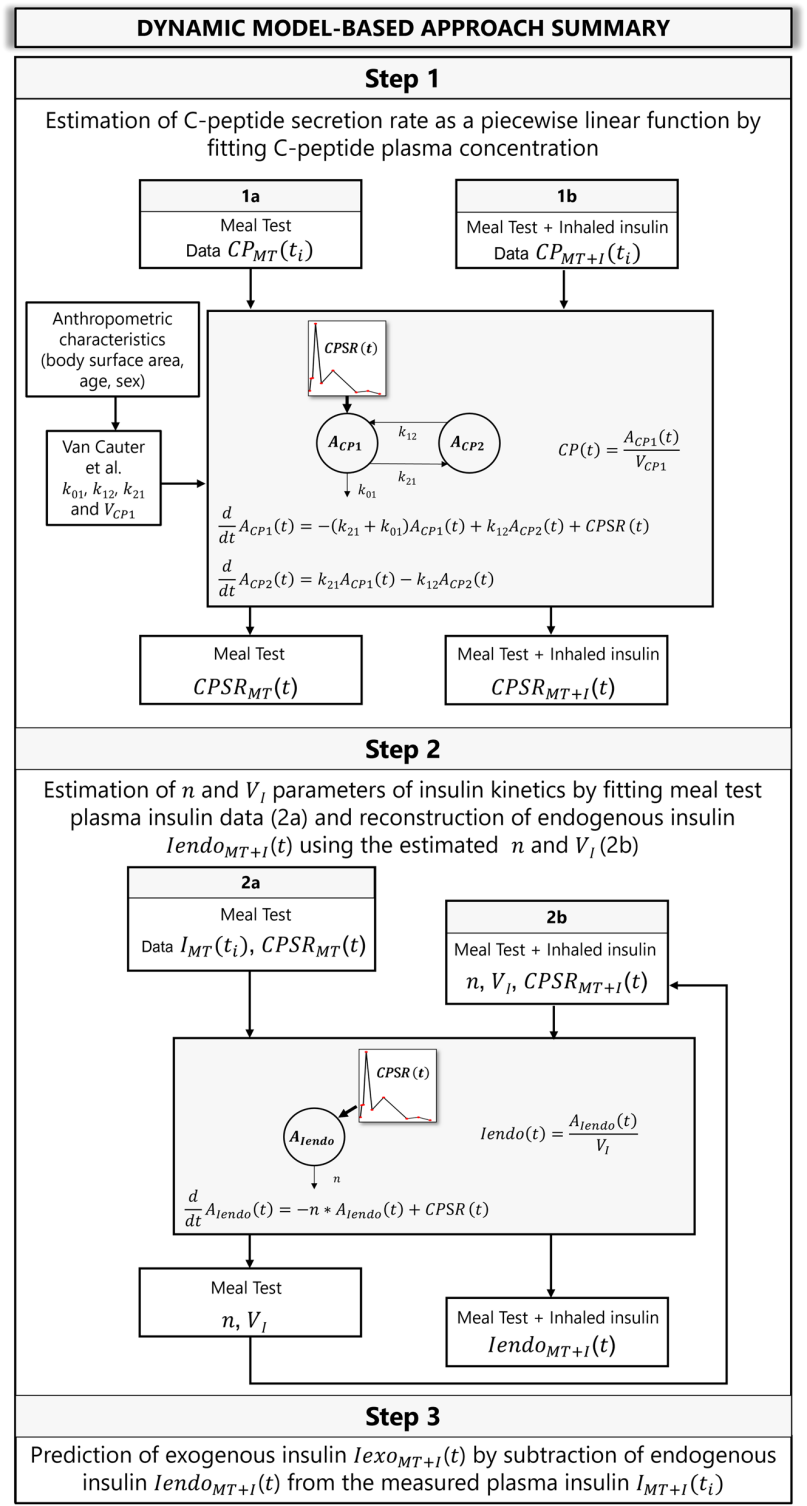
Flow chart of the three-step modeling approach.

The three-step dynamic model-based approach summarized in Fig. 2 was used to estimate the exogenous and endogenous insulin contributions to total plasma insulin.

#### First step

Estimation of the C-peptide secretion rate ($CPSR \, \left(t\right),$ pmol·min^−1^) was performed by solving a deconvolution problem with $CPSR \, \left(t\right)$ as initially introduced by Eaton et al.^21^, with rate constants obtained with method of Van Cauter et al.^22^. Given that C-peptide and insulin are secreted equimolarly, and that C-peptide does not undergo hepatic extraction, insulin secretion rate can be assumed equal to $CPSR \, \left(t\right)$. This approach was applied to the meal test data without (1a in Fig. 2) and with (1b in Fig. 2) prior inhalation of exogenous insulin to obtain ${CPSR \, }_{MT}\left(t\right)$ and ${CPSR \, }_{MT+I}\left(t\right)$, respectively.

#### Second step

The following one-compartment model of insulin kinetics was used to obtain subject-specific estimates of insulin distribution volume ${\text{V}}_{I}$ (L) and elimination rate constant $n$ (min^−1^) using plasma insulin concentration–time measurements^23^:

3 $$\begin{aligned} & \frac{d}{{dt}}A_{{Iendo}} \left( t \right) = - n \cdot A_{{Iendo}} \left( t \right) + CPSR{\mkern 1mu} \left( t \right), \\ & A_{{Iendo}} \left( 0 \right) = CPSR\left( 0 \right)/n \\ \end{aligned}$$

where ${A}_{Iendo}\left(t\right)$ is the amount of plasma insulin (pmol) and $CPSR \, \left(t\right)$ is the insulin secretion rate (pmol·min^−1^). The model predicted endogenous insulin concentration is $Iendo(t)={A}_{Iendo}\left(t\right) /{\text{V}}_{I}$. The model was first applied to the meal test data without prior inhalation of exogenous insulin (2a in Fig. 2). The resulting subject-specific model was then used on meal test data with inhaled insulin to predict the plasma insulin component attributable to endogenous insulin ${Iendo}_{MT+I}\left(t\right)$ (pmol·L^−1^), as depicted in step 2b of Fig. 2.

#### Third step

The contribution of exogenous insulin ${Iexo}_{MT+I}\left(t\right)$ to plasma insulin was obtained by subtracting the predicted endogenous component ${Iendo}_{MT+I}\left(t\right)$ from the total insulin concentration ${I}_{MT+I}\left({t}_{i}\right)$ measured during the meal test with inhaled insulin:

4 $${Iexo}_{MT+I}\left(t\right)={I}_{MT+I}\left({t}_{i}\right) -{Iendo}_{MT+I}\left(t\right)$$

### Model implementation and parameter estimation

Model simulation and parameter estimation were performed using the ADAPT (version 5) software for pharmacokinetic/pharmacodynamic system analysis^24^. The deconvolution problem to estimate $CPSR \, \left(t\right)$ was solved by assuming $CPSR \, \left(t\right)$ as a piecewise linear function, as depicted in Fig. 2, whose parameters (slopes of each line segment) were determined through maximum likelihood estimation using the measured plasma C-peptide measurements. Break points of the piecewise linear function corresponded to the measurement times^25^. Estimation of individual insulin kinetic parameters (${\text{V}}_{I}$ and $n$ in Fig. 2) was obtained using maximum likelihood estimation in ADAPT.

### Comparison with other C-peptide-based methods

The dynamic model-based method for estimating endogenous plasma insulin proposed herein was compared to the “C-peptide correction” methods previously proposed by Owens^13^ and by Marino et al.^14^ These latter two methods attempt to correct the measured insulin concentration by removing the component (assumed constant) attributable to endogenous secretion, thus resulting in the isolated contribution of exogenous insulin.

Application of the method proposed by Owens^13^—also known as “baseline correction”—to the meal test with inhaled insulin data (${I}_{MT+I}\left({t}_{i}\right), {CP}_{MT+I}\left({t}_{i}\right))$ results in the following predicted values of the endogenous ${(Iendo}_{MT+I}\left({t}_{i}\right))$ and exogenous (${Iexo}_{MT+I}\left({t}_{i}\right))$ components of plasma insulin:

5 $${Iendo}_{MT+I}\left({t}_{i}\right)=ICPR\cdot {CP}_{MT+I}\left({t}_{i}\right)$$

6 $${Iexo}_{MT+I}\left({t}_{i}\right)={I}_{MT+I}\left({t}_{i}\right)-{Iendo}_{MT+I}\left({t}_{i}\right).$$

In Eq. (5), $ICPR$ represents the Insulin-to-C-Peptide fractional temporal Ratio and is a constant value throughout the test, computed as follows:

7 $$ICPR=\frac{{I}_{MT+I}\left(0\right)}{{CP}_{MT+I}\left(0\right)}$$

where ${I}_{MT+I}\left(0\right)$ and ${CP}_{MT+I}\left(0\right)$ are the plasma insulin and C-peptide concentrations measured at fasting (i.e., before starting the test), respectively.

The method proposed by Marino et al.^14^, hypothesized the existence of a linear relationship between insulin and C-peptide time courses and exploited a linear mixed effects modeling approach to predict the endogenous component, ${Iendo}_{MT+I}\left({t}_{i}\right)$, and the related exogenous component, ${Iexo}_{MT+I}\left({t}_{i}\right)$, from meal test with inhaled insulin data according to the following equations:

8 $${Iendo}_{MT+I}\left({t}_{i}\right)=ICPR\cdot {CP}_{MT+I}\left({t}_{i}\right)+Intercept$$

9 $${Iexo}_{MT+I}\left({t}_{i}\right)={I}_{MT+I}\left({t}_{i}\right)-{Iendo}_{MT+I}\left({t}_{i}\right).$$

In Eq. (8), $ICPR$ is a constant value throughout the test computed as follows:

10 $$ICPR=Slope+{v}_{subject}$$

where $Intercept$ and $Slope$ in Eqs. (8) and (10) represent the mean values of the intercept and slope in the linear regression analysis across all subjects, thus accounting for the fixed effects; ${v}_{subject}$ is the subject specific deviation from the mean slope and represents the random effects. To estimate parameters of the linear mixed effect model ($Slope, {v}_{subject}$ and $Intercept$), meal test data [${I}_{MT}\left({t}_{i}\right)$ and ${CP}_{MT}\left({t}_{i}\right)$] were used (analogous to Step 2a in Fig. 2).

Each of these C-peptide correction methods was implemented in MATLAB 2019b (The Mathworks); the fitlme built in function was used to perform the required linear mixed effect modeling analysis. Our dynamic model-based estimation method and the two C-peptide correction method results were evaluated by comparing their respective insulin-to-C-peptide fractional temporal ratio values, which for our approach is given by the following equation:

11 $$ICPR({t}_{i})=\frac{{Iendo}_{MT+I}\left({t}_{i}\right)}{{CP}_{MT+I}\left({t}_{i}\right)}$$

### Assessment of the percentage of exogenous insulin dose reaching the plasma

The percentage of initial exogenous insulin dose that reached the plasma (i.e., absolute bioavailability, $\%exo$) was obtained as follows:

12 $$\%exo=\frac{ {{AUC}_{Iexo}}}{{AUC}_{IV}}$$

being ${AUC}_{Iexo}$ (pmol·min/L) the area under the exogenous insulin concentration profile and ${AUC}_{IV}$ (pmol min/L) the area under the curve that would be obtained if the same dose was administered intravenously.

Assuming a single-compartment description, ${AUC}_{IV}$ was computed as follows:

13 $${AUC}_{IV}=\frac{ { V}_{I }\cdot n}{Dose}$$

being ${ V}_{I}$ the insulin distribution volume and $n$ the elimination rate constant estimated in the second step of Fig. 2; $Dose$ is the dose of inhaled insulin administered to each subject (20 or 22 U).

### Statistical analyses

Statistical analysis was performed using the MATLAB 2019b (The Mathworks). The Lilliefors test was used to evaluate if variables come from a normal distribution. Differences in measured glucose, insulin and C-peptide concentrations between meal test and meal test with inhaled insulin were assessed using the nonparametric Wilcoxon signed rank test. The model performance was evaluated in terms of Studentized residuals. Results are presented as mean ± standard deviation unless otherwise designated. Statistical comparisons of the endogenous insulin predicted for both meal test without (${Iendo}_{MT}$) and with (${Iendo}_{MT+I}$) prior inhalation of exogenous insulin were evaluated at each sampling time and in terms of supra-basal area under the curve using the nonparametric Wilcoxon signed rank test. Statistical comparison was also performed by stratifying for gender (male vs. female individuals) and BMI (< 25 vs. ≥ 25 kg/m^2^ classified as normoweight or overweight, respectively).

## Results

**Figure 3:**
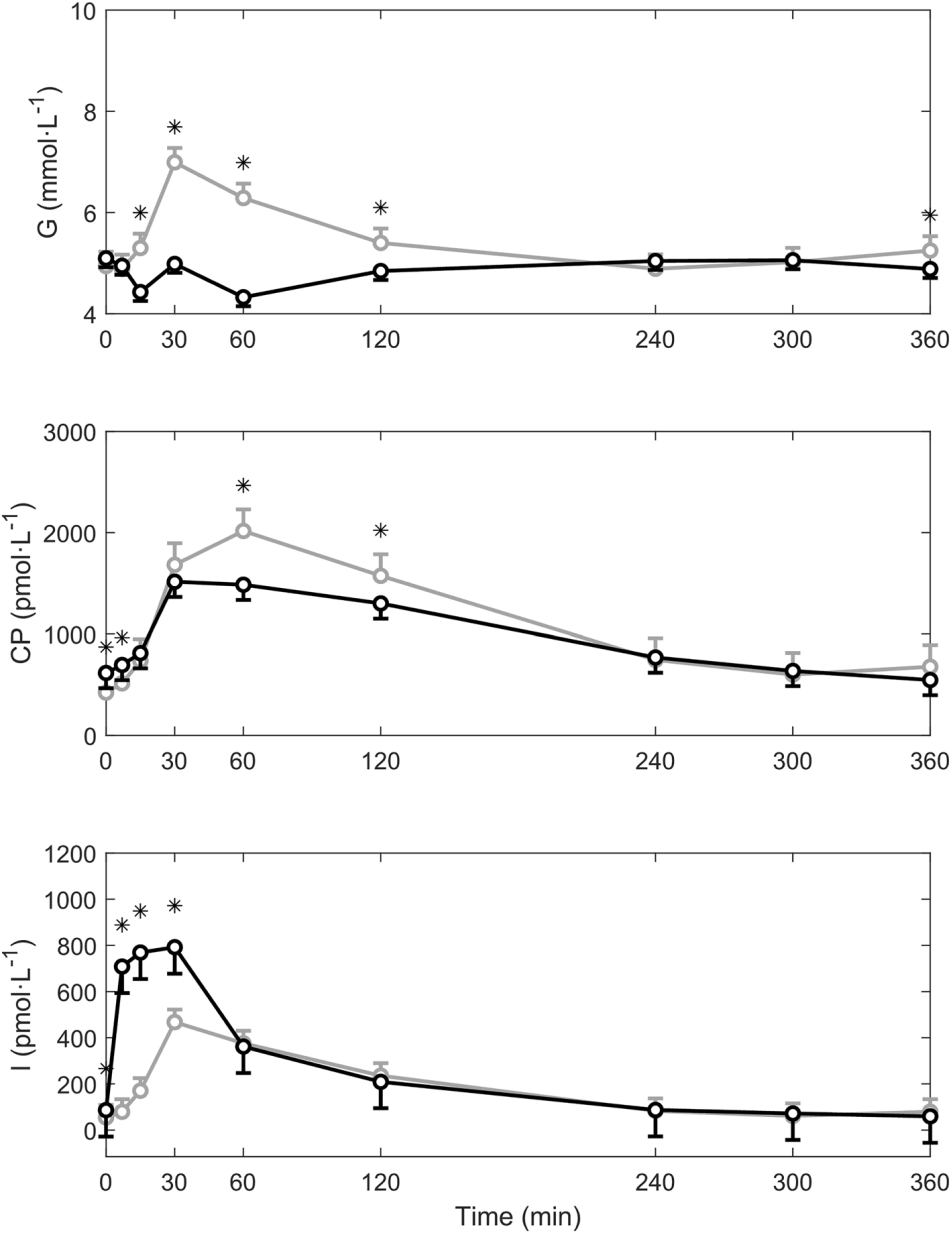
Average plasma glucose (G), C-peptide (CP) and insulin (I) concentrations measured during meal test without (grey line) and with prior inhalation of exogenous insulin (black line). *p < 0.05. Vertical bars represent SE.

The plasma glucose, C-peptide and insulin concentrations measured during the meal test with and without prior inhalation of exogenous insulin are summarized in Fig. 3. The bottom panel of Fig. 3 shows the higher insulin concentration observed in the first hour of the meal test with inhaled insulin. From the central panel, it can be noticed that the observed C-peptide (a marker of endogenous insulin secretion) is lower in the meal test with inhaled insulin during the 30-to-120-min interval following the meal test.

**Figure 4:**
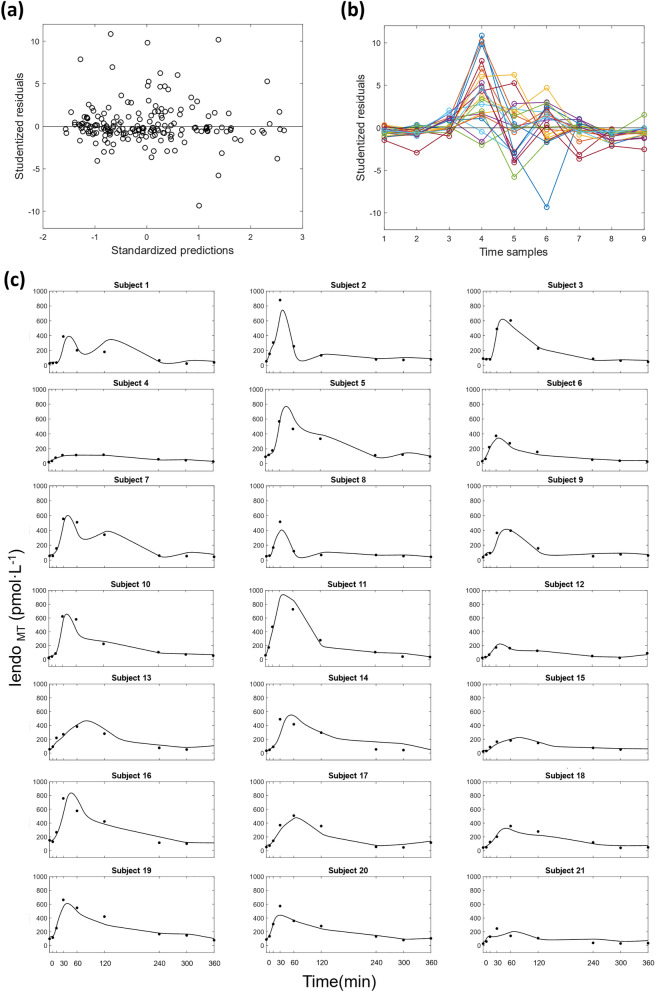
(**a**) Residuals versus standardized predictions; (**b**) residuals versus time samples; (**c**) individual fits for each of the 21 subjects from meal test data; continuous black lines show the model fit; black dots represent meal test measured plasma insulin concentration (pmol·L^−1^).

Estimates for the C-peptide model parameters from Step 1 of the analysis (${\text{V}}_{CP1}$, ${k}_{12}$, ${k}_{21}$, ${k}_{01}$) and for the insulin model parameters from Step 2a (${\text{V}}_{I}$ and $n$) are given in Table 2. Plots of the individual subject’s model predicted plasma insulin concentrations from Step 2a are shown in Fig. 4. Individual subject’s Studentized residuals versus standardized predictions and over time are also shown in Fig. 4.

**Table 2:** Estimates for the C-peptide model parameters from Step 1 of the analysis (mean and standard deviation across subjects) and for the insulin model parameters from Step 2a (mean and standard deviation of individual estimates).

	Mean ± SD
Calculated parameters_Step 1	
${\text{V}}_{CP1}(L$)	4.223 ± 0.350
${k}_{12}$ (min^−1^)	0.050 ± 0.001
${k}_{21}$ (min^−1^)	0.052 ± 0.001
${k}_{01}$ (min^−1^)	0.059 ± 0.001
Estimated parameters_Step 2a	
${\text{V}}_{I}$ (L)	15.680 ± 5.740
$n$ (min^−1^)	0.120 ± 0.050

Data are mean ± standard deviation (SD) unless otherwise indicated.

**Figure 5:**
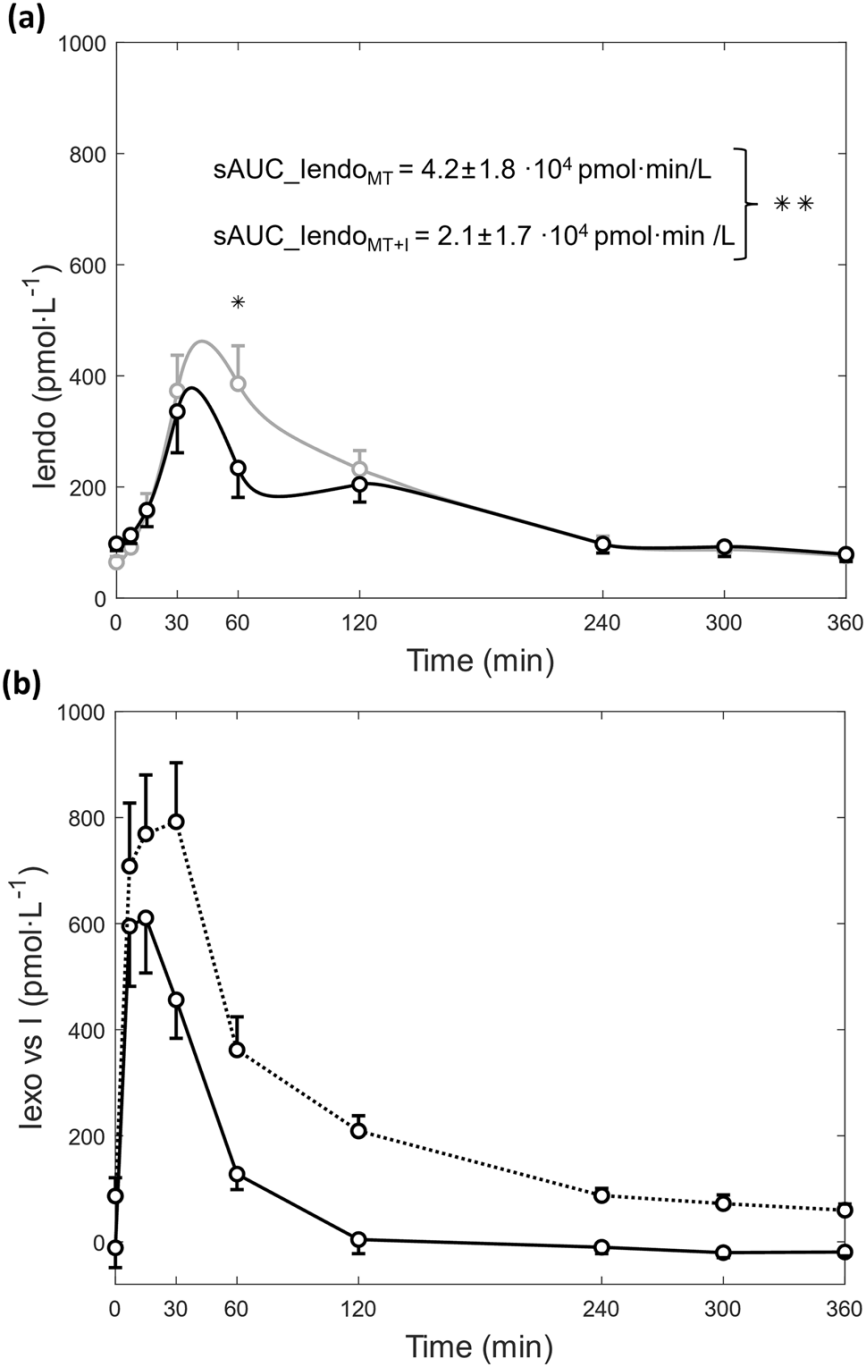
(**a**) Average predicted endogenous insulin component for meal test (${Iendo}_{MT},$ grey line) and meal test with inhaled insulin (${Iendo}_{MT+I},$ black line); *p < 0.01, **p < 0.001; (**b**) average total measured plasma insulin concentration (${Iexo}_{MT},$ dotted line) and average predicted exogenous component (${I}_{MT+I},$ solid line). Vertical bars represent SE.

The endogenous insulin profiles, ${Iendo}_{MT}$ and ${Iendo}_{MT+I}$, predicted based on each subject’s model parameters were averaged and shown in Fig. 5a. A statistically significant difference in endogenous insulin with and without inhaled insulin was found at the 60 min sample time (p < 0.01) and a divergence (accounting for a statistically significant difference according to Wilcoxon signed rank test) in the time course of endogenous insulin between the two tests was present between 30- and 120-min following insulin delivery. Moreover, the supra-basal area under the curve of ${Iendo}_{MT+I}$ was significantly lower than that of ${Iendo}_{MT}$ (sAUC_${Iendo}_{MT+I}=2.1\pm 1.7\times$10^4^ pmol∙min/L, sAUC_${Iendo}_{MT}$ = 4.2 ± 1.8 × 10^4^ pmol∙min/L, p < 0.001). For the meal test with inhaled insulin, the predicted exogenous insulin contribution to the total plasma insulin is shown in Fig. 5b and accounts for on average 34 ± 18% of total plasma insulin. Insulin-to-C-Peptide fractional temporal Ratio ($ICPR$) obtained from the dynamic model-based method ranged from 0.13 ± 0.01 to 0.22 ± 0.02 (with the peak observed at 30 min), and falling between the $ICPR$ obtained with the baseline correction method (0.16 ± 0.05) and that obtained with the linear mixed effect method (0.23 ± 0.01). The percentage of initial exogenous insulin dose that reached the plasma, $\%exo$, resulted to be 42 ± 21% ranging from a minimum of 11% to a maximum of 77%.

When considering the meal test only, total insulin (time course and area under the curve) did not significantly differ in the subgroups, and no significant difference was detected in any of the time samples in endogenous insulin time course in male vs. female and in normal weight vs. overweight comparison. However, a tendency to significance was observed in the differences between males and females when considering the suprabasal area under the endogenous insulin curve (3.4 ± 1.5 × 10^4^ vs. 4.9 ± 1.8 × 10^4^ pmol∙min/L, p = 0.057); this difference became significant when considering the meal + inhaled test (1.1 ± 1.2 × 10^4^ vs. 3.0 ± 1.7 × 10^4^ pmol∙min/L, p = 0.01). During the test, no significant difference was observed in the exogenous component (time samples and area under the curve). As for the estimated model parameters, an expected significant difference was observed in the distribution volume between normoweight and overweight individuals (12.5 ± 4.5 vs. 18.6 ± 5.6 L, p = 0.01).

## Discussion

### Novelty, relevance, and clinical applications

A general model-based methodology has been developed that allows for quantification of the separate contributions of exogenous and endogenous insulin to total peripheral plasma insulin concentration following insulin administration. As first element of novelty, the methodology does not require complex experimental procedures to suppress the endogenous component as those traditionally used and can be applied to tests performed in more physiological conditions (i.e., meal tolerance test). Secondly, the procedure allows an easier quantification of percentage of exogenous insulin dose reaching the plasma, a concept usually indicated as absolute bioavailability and regarded as a pharmacokinetic feature of the drug. Determination of this feature usually requires a complex experimental setup, including intravenous drug administration and the suppression previously mentioned; however, the proposed approach not only overcomes the limitations of the traditional experimental setup but also facilitates determination of intra- and inter-individual variability in absolute bioavailability, thus broadening the perspective with respect to the potential of bioavailability in the direction of “individual bioavailability”. Eventually, the approach can be implemented in an easy-to-use integrated software tool for quantifying the exogenous component of systemic plasma insulin and the individual bioavailability, in an effort to assess patient-specific dose–response following insulin administration during a meal test.

Application of the proposed method to a clinical trial based on inhaled insulin administration in healthy subjects provided insight on the difference in endogenous contribution to plasma insulin following meal tests with and without inhaled insulin (see Fig. 5a). Indeed, the predicted time course of endogenous insulin showed a 18.2% reduction in its peak value and the overall endogenous insulin exposure, as assessed by the supra-basal area under the endogenous insulin curve, was reduced by a factor of two following inhaled insulin administration. This general action of exogenous insulin to reduce endogenous insulin secretion, which can be attributed to a negative-insulin-feedback loop mechanism, has been reported previously for other exogenous insulin formulations^26–28^ and may contribute to preserving beta-cell function in patients with type 2 diabetes^29^ and also type 1 diabetes^30^. The ability to separately quantify exogenous and endogenous contributions with their own dynamic behaviors while capturing the effect that one has on the other may open new scenarios in the field of precision medicine by designing tailored therapeutic regimens on the basis of the individual's capability to manage changes in blood glucose^31–34^. Indeed, evidence showed the existence of a log-linear relationship between glycemic variability, a measure of glycemic control, and endogenous insulin secretion in insulin-treated and non-insulin-treated patients with type 2 diabetes^35^. However, studies were conducted exploiting fasting C-peptide as a marker of endogenous secretion and consideration of the time course of endogenous insulin, as done in the present study, may help to investigate how target trends in glucose response can be obtained and to modulate exogenous insulin doses accordingly. As for the case of inhaled insulin, there is evidence that Technosphere Insulin is beneficial to reduce daytime glycemic variability^36^, thus the method proposed herein could be useful to elucidate the role of endogenous insulin in mediating this relationship.

The present method also allows investigating how the ability of exogenous insulin in modulating endogenous secretion differs among different categories of individuals. A preliminary investigation performed stratifying the study cohort in relation to body mass index and gender showed a significance difference between males and females when considering the suprabasal area under the endogenous insulin curve and at the same time no significant difference in the exogenous profile, possibly indicating similar absorption capability. From these results it is not possible to draw definitive conclusions due to limitations in the sample size, but the difference in the endogenous insulin secretion observed between male and female individuals is in agreement with results of previous studies^37^.

Quantification of individual bioavailability obtained by the present approach may be also framed in the domain of personalized therapy; indeed, attempts have been recently made to design tools based on personalized bio-impedance modelling for real-time monitoring of the amount of bioavailable insulin, with the aim to achieve a more accurate insulin administration accounting for both the intra- and inter-individual variability in insulin bioavailability^38,39^. Range of values of bioavailability obtained by the approach proposed in the present study furtherly supported the fact that a high variability exists among individuals and tools for real-time monitoring of the amount of bioavailable insulin may benefit from such an approach to improve their reliability (as for example, in calibration procedures).

### Comments on the applied methodology

The proposed method for decomposing total plasma insulin into its endogenous and exogenous components involves the use of compartment models to describe the kinetics of C-peptide (Step 1) and the kinetics of insulin (Step 2). In contrast to other methods that attempt to determine insulin secretion using measured plasma insulin alone^40^, our approach also requires measurement of associated C-peptide concentrations whose assessment in clinical practice is increasingly encouraged^41^. For C-peptide, the well-established two compartment model used by Eaton et al.^21^ was adopted, with specific parameters determined based on each subject’s body surface area, age and sex following Van Cauter’s method^22^, extensively exploited for similar purposes. It is correct that other factors contributing to inter subject differences are not taken into account. On the other hand, determining unique subject specific C-peptide kinetic parameters would require complex experiments designed to estimate each subject’s C-peptide kinetics. To represent the C-peptide secretion rate profile, a piece-wise linear function was assumed with the break points corresponding to the measurement times, which allows easy estimation of the unknown slopes of each line segment using the measured C-peptide concentration data. The choice of piece-linear approximation to the C-peptide release rate has advantages over fully parametric methods that use a single overall function (e.g., Gaussian^42^) to represent C-peptide release rate. Moreover, despite we recognize that nonparametric deconvolution approaches could have some advantages (i.e., finer input discretization grids), we noted that the simplified approximation to the C-peptide release rate used in this work can adequately describe the time course of observed insulin data when used as the input to the insulin kinetic model, as shown in Fig. 4. A one-compartment model was then used to describe plasma insulin kinetics, parameterized in terms of distribution volume and rate constant of elimination, with the C-peptide secretion rate function determined in Step 1 serving as the input. The use of single compartment to represent insulin kinetics was deemed appropriate during a meal test, where insulin levels remain low enough to avoid possible non linear processes (such as for instance receptor saturation) and hence maintaining linear dynamics^43^.

To maintain our approach the simplest possible, we did not include explicitly hepatic insulin extraction. Nonetheless, the elimination rate constant, $n$, represents the overall measure of insulin clearance^23,44^ which can account for the potential differences between healthy subjects and subjects with diabetes^23^. Moreover, hepatic insulin extraction does not exhibit high temporal variability during meal tests, therefore no particular model for this process is necessary. The resulting estimates of the elimination rate constant (Step 2a) are in good agreement with those reported in other studies involving healthy subjects^23,44,45^, further supporting the choice of C-peptide function approximation and insulin kinetic model. The individual subjects’ estimates of both insulin distribution volume and elimination rate constant obtained from the meal test alone were assumed to be the same in each subject during the meal test with inhaled insulin. These estimates were then used in such meal test with inhaled insulin to determine the endogenous contribution to plasma insulin (Step 2b), and subsequently the exogenous component (Step 3).

As for the bioavailability estimation, to overcome the need to perform a second experiment involving intravenous insulin administration, we exploited each subject insulin pharmacokinetic estimated parameters in order to compute a surrogate area under the curve that would have been obtained if the same dose would have been injected intravenously. This assumption has been deemed appropriate since the insulin kinetic parameters are estimated independently from the route of administration, being the description of the absorption bypassed.

### Comparisons to other C-peptide based methods

The dynamic model-based methodology presented herein addresses some of the limitations of previously reported approaches for separating exogenous and endogenous contributions to plasma insulin following insulin administration. In the “baseline correction” or Owens method^13^, the endogenous contribution to plasma insulin is calculated to be a constant fraction of total insulin based on a single baseline insulin sample, and thus ignores the systems dynamics included in Steps 1 and 2 in our proposed modeling framework (Fig. 2). Indeed, a recent study that evaluated C-peptide baseline correction method concluded that in the presence of unsuppressed endogenous insulin caution should be taken when using such methods for predicting exogenous insulin^46^. In contrast, the method proposed by Marino et al.^14^ exploiting linear mixed effects modeling partially overcomes the limitations of the baseline correction method by considering measurement time points over the whole test. This method, however, again ignores the insulin dynamics included in Step 2 of our approach, as pointed out by the resulting differences in the three methods in terms of insulin-to-C-peptide fractional temporal ratio between the endogenous insulin component and the measured C-peptide. Results from the baseline correction and linear mixed effect methods indicate that they maintain a constant relationship throughout the test. The prediction obtained with the dynamic model-based method, in contrast, results in a time varying insulin-to-C-peptide fractional temporal ratio, which better reflects insulin dynamics; in this case, a fast change (rapid increase followed by rapid decrease) is observed in the first hour of the test.

It has to be acknowledged that comparison has been limited to methods relying on C-peptide, although other methods based on “glucose correction” have been proposed^11^. The reason for this choice relies on the fact that methods based on glucose were developed in applications devoted to glucose control, thus in conditions in which additional simplifying assumptions are needed.

### Limitations and related comments

The study design required with the proposed method has some limitations. Primarily, the application of the proposed approach requires to perform two different meal tests, one with and one without inhaled insulin, which would need to be conducted so as to minimize any carryover effects, i.e., the influence of the first test on the second one; however, other methods based on meal test have the same requirements^14^. Moreover, the method has been evaluated in a relatively limited number of healthy subjects, and evaluation in diabetic patients is needed. Furthermore, another issue has to be acknowledged: indeed, it has to be noted that a direct evaluation of the quantities predicted by the model-based approach under experimental conditions similar to physiological ones (i.e., meal test), however, cannot be achieved by using Technosphere^®^ Insulin. This is because, being its formulation based on regular human insulin, it is indistinguishable from that secreted endogenously. In future studies, this direct assessment could be evaluated across other insulin analogs that are distinguishable from regular human insulin by using assays based on specific monoclonal antibodies that have low cross-reactivity to insulin analogs but high reactivity to endogenously produced insulin. Nonetheless, as a proof of reliability of the proposed method, it can be noted that the predicted exogenous insulin time course shown in Fig. 5b is qualitatively comparable, in terms of onset and duration, to that previously reported for a dose of 24U of inhaled insulin^16^. Similarly, the time course is characterized by a fast onset (peak around 15 min) and a relatively short duration (baseline reached after 120 min, Fig. 5b); however, strict comparison is not possible since in this latter study a less refined method was used to quantify exogenous insulin. Lastly, the estimates here obtained for absolute bioavailability are not directly comparable to those previously reported in the literature for the case of Technosphere^®^ insulin (which provided lower values, usually not exceeding 15%^9,47,48^) for multiple reasons; indeed, previous estimates were obtained exploiting the baseline correction method, which could have produced underestimation of the exogenous insulin profile; moreover, in previous studies insulin was administered using different inhalation devices, which may be less efficient than the one here used^16^.

## Conclusions

A dynamic model-based approach has been developed to separate exogenous and endogenous insulin contributions to total plasma insulin during a meal. Application to the case of inhaled insulin showed an effect of exogenous insulin on the endogenous secretion, which can be potentially beneficial to preserve the latter. This methodology could be useful to assess the exogenous insulin profile in studies based on subjects with non-negligible endogenous insulin and, when introduced in the framework of personalized therapy, to provide patient-specific dose–response following insulin administration. Finally, we noted that although exogenous insulin in this study was provided through inhalation, the proposed approach can be applied to other routes of administration.

## Data Availability

Data are available from G.P. (giovannipacini49@gmail.com) and/or from the corresponding author (m.morettini@univpm.it), upon reasonable request and with permission of Mannkind.
